# Runs of homocigosity and its association with productive traits in Mexican Holstein cattle

**DOI:** 10.1371/journal.pone.0274743

**Published:** 2022-09-19

**Authors:** José G. Cortes-Hernández, Felipe J. Ruiz-López, Carlos G. Vásquez-Peláez, Adriana García-Ruiz

**Affiliations:** 1 Programa de Maestría y Doctorado en Ciencias de la Producción y de la Salud Animal, Universidad Nacional Autónoma de México, Ciudad de México, México; 2 Centro Nacional de Investigación Disciplinaria en Fisiología y Mejoramiento Animal, Instituto Nacional de Investigaciones Forestales Agrícolas y Pecuarias, Ajuchitlán Colón Querétaro, México; 3 Departamento de Genética y Bioestadística de la Facultad de Medicina Veterinaria y Zootecnia, Universidad Nacional Autónoma de México, Ciudad de México, México; University of Life Sciences in Lublin, POLAND

## Abstract

The objective of this study was to describe the runs of homozygosity (ROH) detected in the Mexican Holstein population and to associate them with milk, fat and protein yields, and conformation final score. After imputation and genomic quality control, 4,227 genotyped animals with 100,806 SNPs markers each were used. ROH with a minimum length of 1 Mb and a minimum of 10 SNPs were included in the analysis. One heterozygous SNP marker and five missing genotypes per ROH were allowed. A total of 425,098 ROH were found in the studied population (71.83 ± 10.73 ROH per animal), with an average length and coverage of 4.80 ± 0.77 Mb, and 276.89 Mb, respectively. The average chromosome length covered by ROH was 10.40 ± 3.70 Mb. ROH between 1 and 2 Mb were the most frequent in the population (51.33%) while those between 14 and 16 Mb were the least frequent (1.20%). Long chromosomes showed a larger number of ROH. Chromosomes 10 and 20, had a greater percentage of their length covered by ROH because they presented a largest number of long ROH (>8 Mb). From the total ROH, 17 were detected in 1,847 animals and distributed among different chromosomes, and were associated with milk, fat and protein yield and percentage, and conformation final score. Of the ROH with effects on production traits, the majority were found with a length between 1 and 4 Mb. These results show evidence of genomic regions preserved by genetic selection and associated with the improvement of the productivity and functionality of dairy cattle.

## Introduction

Runs of Homozygosity (ROH) are contiguous strands of homozygous DNA present in individuals due to their ancestors transmitting identical haplotypes from one generation to the next, which represents the genomic homozygosity produced by mating genetically related individuals [1]. Kim et al. [2] mentioned that ROH associated with harmful recessive alleles can show regions that affect the physical condition of individuals; which in most cases, result from a strong artificial selection directed towards few traits using a reduced number of animals. Rebelato et al. [3] suggested that ROH can be used for the genomic characterization of populations and the identification of specific patterns of breeds, known as signatures of selection or fingerprints.

ROH detection was first performed in humans in the late 90’s, and since then, ROH are considered a reliable source of information for studying the history of human population and decoding the genetic basis of complex monogenic traits and diseases [4]. In cattle, the development of molecular methods has made possible the identification of chromosomic regions, genes and allelic variants associated to economically important traits, as milk yield, meat production, growth and reproduction [5].

The Holstein breed has dominated the dairy industry for many years. The adoption of reproductive technologies, the selection intensity and the use of a limited number of sires with high genetic merit, have caused an increase of inbreeding, which increased the possibility of finding identical ROH by descent [6].

The length of homozygous segments depends on the degree of shared parental ancestry. ROH caused by recent inbreeding (up to 5 generations) tend to be longer (> 10 Mb) because those have fewer opportunities for recombination to break apart segments that are identical by descent [2]. ROH from older origin (around 50 generations) generally are shorter (1 Mb), because chromosomal segments have been broken down by repeated meiosis [7]. However, it is necessary to consider the differences in recombination rates in specific regions of the genome, because some long segments could persist [8].

Nowadays, several studies have shown methodologies to calculate the inbreeding coefficient of an individual through the ROH, quantifying the proportion of the genome covered by ROH [9–11]. These methods can be used to calculate how recent or old the inbreeding is in the population and give a real proportion of the genome that is homozygous [12].

Different studies [13, 14] have shown ROH associated with economically important traits as a result of the high use of selected families or individuals in populations. A study performed in Holstein cattle [14], reported a higher number of ROH in chromosomes (BTA) 13, 20 and 26, and lower numbers on BTA 1, 2, 7 and 16. This suggests that DNA conservation is not similar throughout the genome and the ROH distribution is influenced by the different selection strategies through the years [15].

ROH reported on chromosome 12 have been associated with genes related to infectious diseases, mastitis and locomotor disorders in Holstein cows. A long region of homozygosity associated with genes related to metabolic syndromes, infectious and reproductive diseases has been detected on chromosome 15, revealing complex relationships between immunity, metabolism and functional disorders, while other ROH that contain genes associated to these diseases have also been found on BTA 3, 5, 7, 13, and 18 [16]. In a genome wide association study which included 45,753 SNPs, Pryce et al. [13] detected ROH (>3 Mb) associated with a decrease of up to 12.5 days of calving interval and of 260 liters of milk yield.

Pryce et al. [13] and Ouborg et al. [17] suggest that identification of ROH in the genome and their association with many traits, help improve the selection process and decrease inbreeding depression.

Therefore, the objective of this study was to identify ROH, describe them and identify their association with productive traits in Holstein cattle from Mexico.

## Material and methods

A total of 5,918 genotypes of Mexican Holstein cattle were used, which were distributed in 36 herds of 10 states of the country and each animal included 100,806 SNPs markers. Quality control excluded animals with call rates <0.90 and SNP with minor allele frequencies <0.02, or with a call rates <0.90, or a Hardy Weinberg P value <0.0001 [7, 16].

To define the ROH, homozygosity regions with a minimum length of 1 Mb with at least 10 SNP were included. With these parameters, the risk of including very short ROH was avoided, a condition that is very common due to linkage disequilibrium (LD) [1]. LD is associated with the presence of linked genes, genes jointly inherited transmitting from parents to sons, affecting the recombination frequency (<50) [18]; in addition, 1 heterozygous SNP and 5 missing genotypes were allowed per ROH [8].

The ROH analyses was done using the SNP & Variation Suite v8.3 [19], while the analyses of the obtained data were carried out with SAS 9.3 [20] and R 3.6.1 software [21].

For the association analysis the phenotypes were the predicted transmitting abilities (PTA) of milk, fat, protein yield and conformation final score; information provided by the National Center for Disciplinary Research in Physiology and Animal Improvement of the National Institute for Forestry, Agriculture and Livestock Research (INIFAP) [22]. The association analyses were performed with a linear regression of the PTA on the ROH [2, 11] with the SNP & Variation Suite v8.3 [19] and the ARS-UCD1.2 [23] database was used as a reference for searching annotations related with the regions found in this study.

To estimate the inbreeding coefficient from ROH (Froh), the methodology used was that proposed by Mcquillan [9].

To study the effect of ROH on milk, fat, protein, somatic cell score and conformation final score, the records of animals in first lactation adjusted to 305 days were selected [24]. The ROH were classified in six length classes: from 1–2 Mb, 2–4 Mb, 4–6 Mb, 6–8 Mb, 8–16 Mb and >16 Mb. The following linear model was used for each trait and class of ROH separately.


$$Y_{ijk}=\mu +{HY}_{i}+{month}_{i}+{\alpha *age}_{k}+\beta_{1}*{Froh}_{k}+\sum_{l=1}^{n}\beta_{2}*{Lroh}_{kl}+{cow}_{k}+e_{ijk}$$

Where *y*_*ijk*_ is the trait evaluated, *μ is the general mean*, *HY*_*i*_ is the *ith* herd-year of calving (119 classes), *month*_j_ is the *jth* month of calving (12 classes), *α* is the regression coefficient for *age* at calving for the *kth* cow (*age*_*k*_), *β*_*1*_ is the regression coefficient for *Froh*_*k*_, which was the inbreeding coefficient calculated from ROH for the *kth* cow, *β*_*2*_ is the regression coefficient for *Lroh*_*kl*_, which represents the presence of a particular ROH (*Lroh*_*kl*_ = 1 if *roh*_*l*_ is present in the *cow*_*k*_ and 0 if isn’t) within length class, *cow*_*k*_ is the random genetic effect for the *kth* cow, and *e*_*ijk*_ is the random error term.

The linear model analyses were carried out using the BLUPF90 program [25]. The effect of ROH on a given trait was assessed based on the significance of its associated regression coefficient (β_2_) using the t-statistic [26].

## Results

The average length of ROH in the population was 4.80 ± 0.77 Mb, with a maximum and minimum lengths of 100.57 Mb and 1.55 Mb, respectively, and the number of ROH per animal ranged from 12 to 175, with an average of 71.83 (Table 1) and the Froh percentage calculated in the studied population was of 2.77 ± 0.71.

**Table 1:** Average length and SNP number per ROH, and number of ROH per animal in the Mexican Holstein population.

	Mean	Máximum	Mínimum	Std. Dev.
**Length in Mb**	4.80	100.57	1.55	0.77
**SNP/ROH**	146.95	4371.00	6.00	201.08
**ROH/Animal**	71.83	175.00	12.00	10.73

Mb: Megabases, ROH: Runs of homozygosity, SNP: Single Nucleotide Polymorphism, Std. Dev: Standard Deviation.

The most common ROH were those with lengths from 1 to 2 Mb, which represented 52.01% (221,086 ROH), followed by the ones with a length of 2 to 4 Mb with 22.02% (93,596 ROH). The less frequent ones were those of 14 to 16 Mb, with 5,029 (1.18% of the total ROH). ROH with a length greater than 16 Mb, represented 3.48%, finding some of up to 100.57 Mb, with an average length of 23.29 Mb for this category (Table 2).

**Table 2:** Proportion of ROH per length.

Length (Mb)	Number	Percentage	Average length (Mb)
**1 to 2**	221,086	52.01	1.35
**2 to 4**	93,596	22.02	2.79
**4 to 6**	38,811	9.13	4.89
**6 to 8**	22,518	5.30	6.89
**8 to 10**	13,426	3.16	8.94
**10 to 12**	9,271	2.18	10.92
**12 to 14**	6,549	1.54	12.93
**14 to 16**	5,029	1.18	14.92
**>to 16**	14,812	3.48	23.29
**TOTAL**	425,089	100	

**Fig 1:**
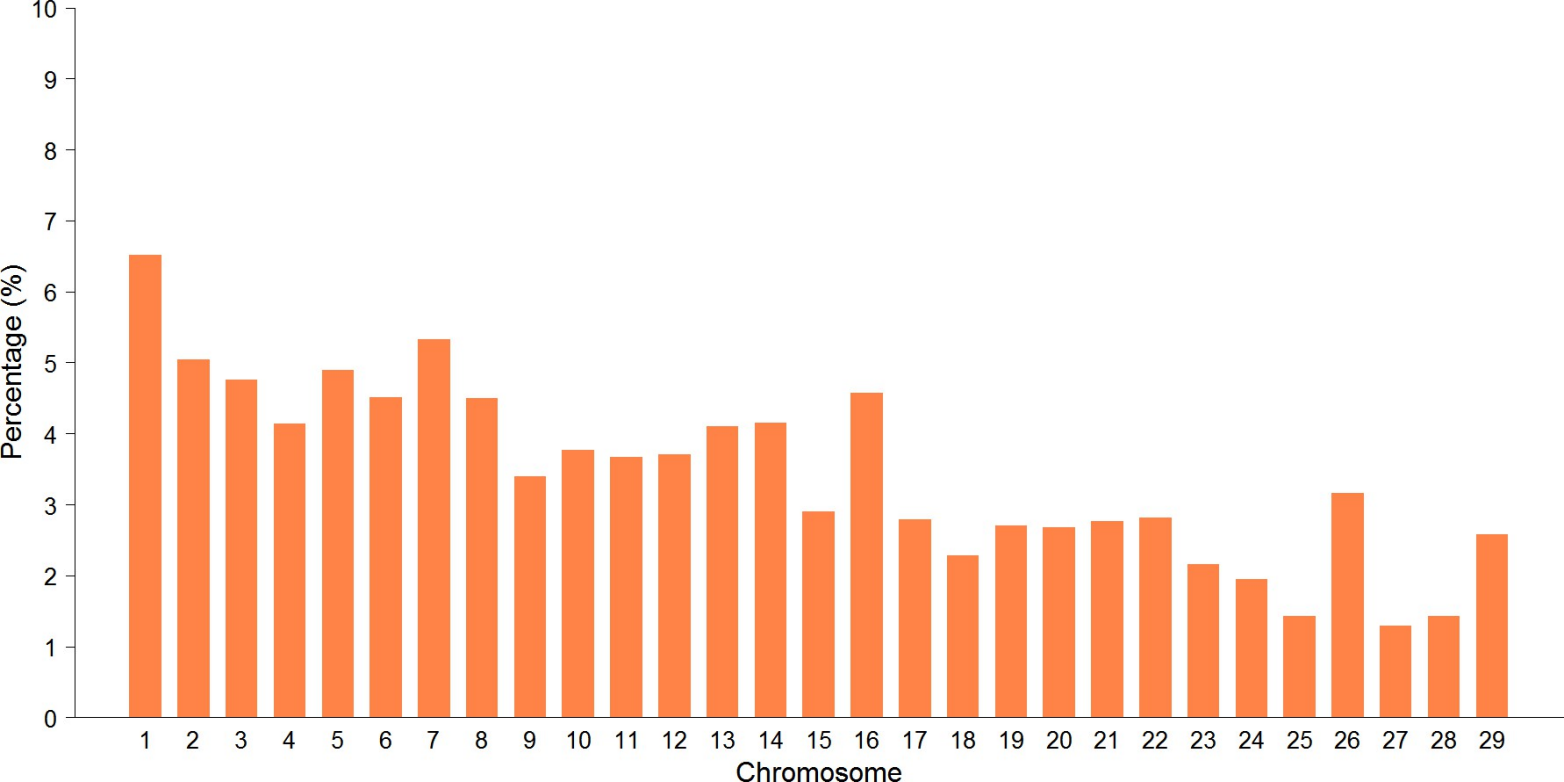
ROH percentage by chromosome in the Mexican Holstein population.

From the total ROH detected in the studied population, 119,973 were unique (only present once in the population) and 305,125 were repeated (≥2). Given that the percentage of the length of each chromosome included in a ROH is similar across chromosomes, it is expected that longer chromosomes would have higher percentages of the total ROH length. Chromosome 1, the longest in cattle with ~158.34 Mb in length, presented the highest percentage of ROH, with 6.52% (27,711 runs, Fig 1), with an average in length of 3.80 Mb. The shortest BTA presented the lowest number of ROH; 13,448 ROH in BTA 26, with an average length of 3.02 Mb, which represents 3.16%; 6,054 ROH (1.42%) were detected on chromosome 28 with an average length of 3.81 Mb, and 6,067 were identified on chromosome 25 (1.43%) with an average length of 3.69 Mb.

**Fig 2:**
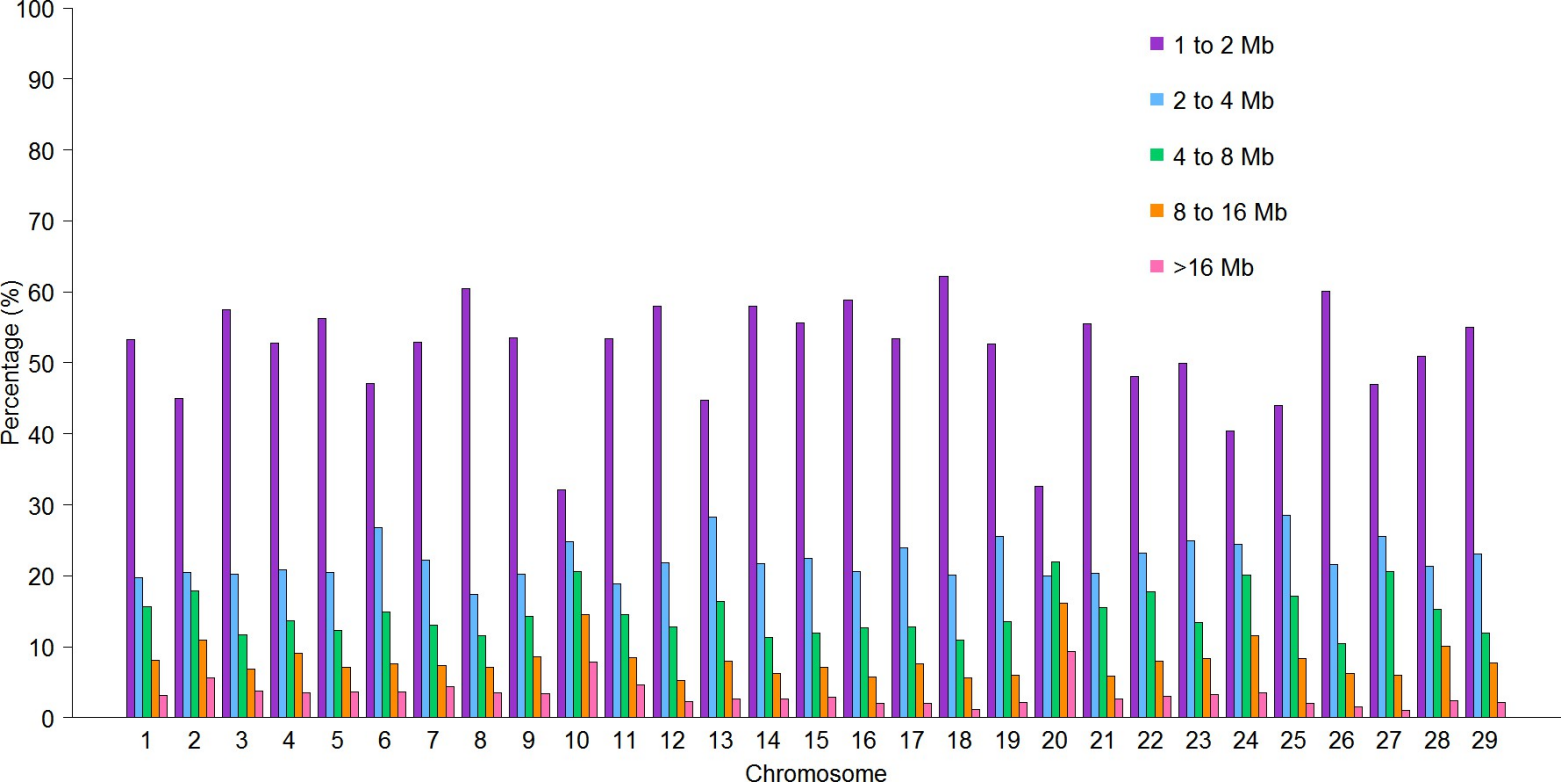
Proportion of ROH, classified by length in each chromosome.

Fig 2 shows the percentage of ROH classified by length in each chromosome, BTA 10 and 20 presented the highest percentage of longer ROH, with 14.81% and 16.38% of ROH respectively from 8 to 16 Mb, and 7.93% and 9.38% respectively of ROH >16 Mb.

**Fig 3:**
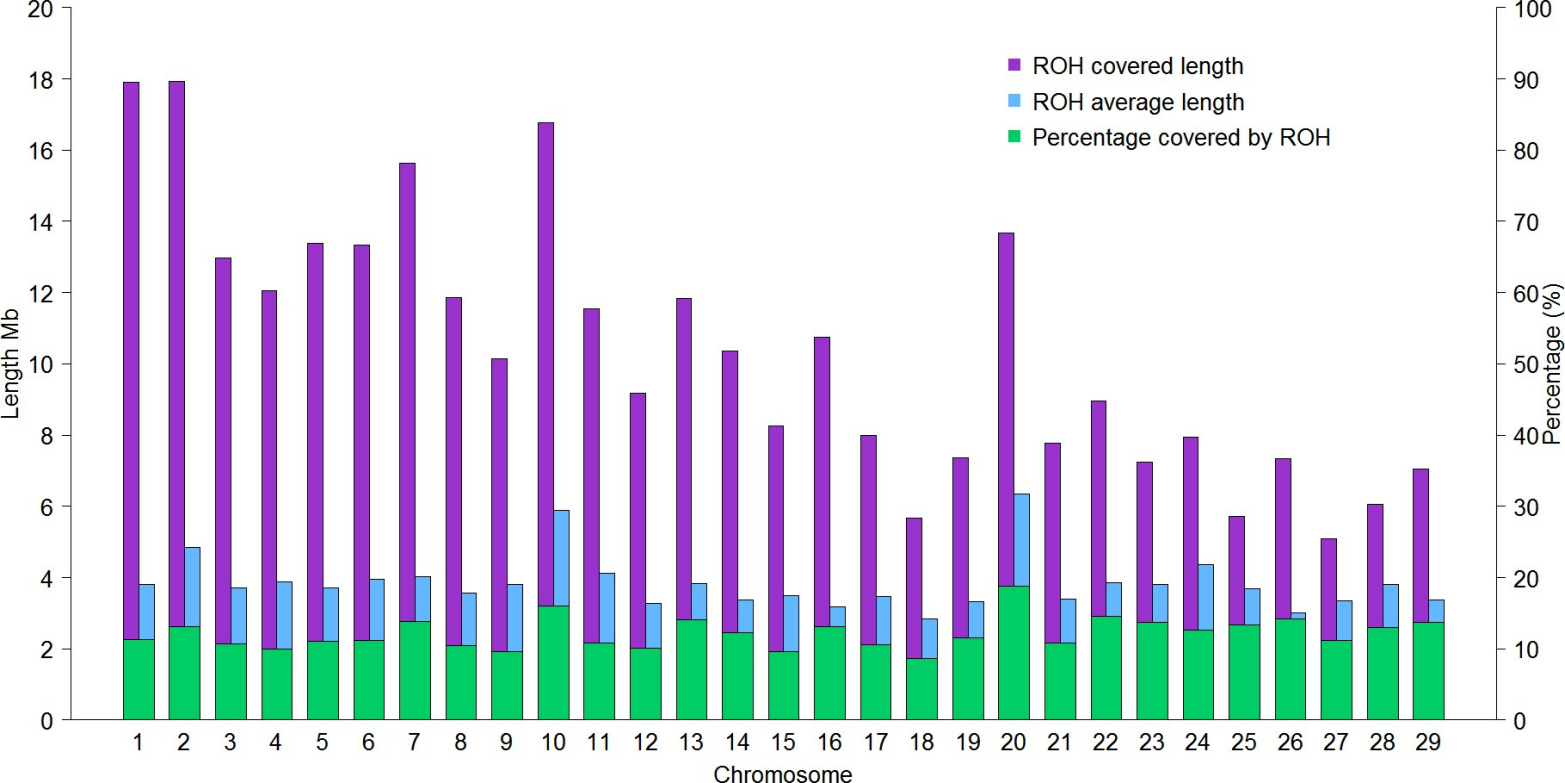
Length, average length and percentage of the genome covered by ROH in each chromosome in the Mexican Holstein population.

Fig 3 shows the proportion of each chromosome covered by ROH, displaying that BTA 10 and 20 had the highest percentage of length covered by ROH 16.07% and 18.78% respectively. BTA 25, the shortest in cattle (42.90 Mb in length) presented a proportion covered by ROH of 13.31% and BTA 1 the longest of this species presented a proportion of 11.31%.

### Association of ROH with productive traits; milk, fat, protein, somatic cell score and conformation final score

The mean of milk production in first lactation adjusted to 305 days for the cows in this study was 12,743.03 ± 2,198.95.kg, for protein the mean was of 356.02 ± 129.94 kg and the mean for fat was of 385.99 ± 144.89 kg. The mean of conformation final score was of 80.46 ± 2.83, which qualifies a cow as good-plus.

Table 3 shows the significant ROH associated with productive and conformation traits, as well as the sample percentage in which they were present in the study. In BTA 26, ROH of 1.09 Mb were found in 20.65% of the population studied, with positive effects on milk (+60.51 kg) and negative effects on somatic cells (-0.08).

**Table 3:** Effect of ROH on productive traits; milk, fat, protein, somatic cell score and conformation final score.

BTA	% Population	Start position (Mb)	End Position (Mb)	Length (Mb)	SNPs	EFFECT
8	10.07	61.51	62.54	1.03	18	-14.13 Kg of milk fat.
7	13.57	46.97	48.01	1.04	23	+350.53 kg of milk yield and +8.86 kg of milk protein.
14	9.80	24.26	25.33	1.07	61	-0.79 points of conformation final score.
1	8.36	21.29	22.36	1.07	49	-0.12 of protein percentage.
5	16.09	78.96	80.03	1.07	31	-0.11 of fat percentage.
2	11.29	78.06	79.14	1.08	36	-0.08 of protein percentage.
26	20.65	19.17	20.26	1.09	29	+60.51 kg of milk yield and -0.08 points in somatic cell score.
8	10.94	110.82	111.91	1.10	21	+268.67 Kg of milk yield, +11.48 kg of milk fat and +8.20 kg of milk protein.
8	9.09	95.71	96.86	1.14	24	+0.12 points in somatic cell score.
1	10.91	83.84	85.15	1.31	39	-0.07 protein percentage.
16	8.41	72.02	73.43	1.41	36	+0.16 points in somatic cell score.
13	9.53	0.36	1.88	1.52	63	+299.02 kg of milk yield and -0.12 points in somatic cell score.
2	13.95	84.54	86.09	1.55	52	-0.18 points in somatic cell score.
6	9.06	9.52	11.60	2.07	86	+0.97 points in conformation final score.
16	4.88	74.30	76.45	2.15	69	-0.21 protein percentage.
6	7.49	68.97	71.25	2.29	101	+483.27 Kg of milk yield.
7	6.16	102.90	105.26	2.37	82	+13.51 Kg of milk fat and +0.17 points in somatic cell score.
4	4.21	42.61	45.08	2.47	92	+0.12 protein percentage.
16	5.24	57.83	60.35	2.52	107	+0.83 points in conformation final score.
2	4.26	45.91	48.62	2.70	109	+711.42 kg of milk yield, +18.32 kg of milk fat and +18.37 kg of milk protein.
21	5.02	4.61	8.19	3.58	122	+0.10 protein percentage.
20	3.36	34.90	39.18	4.27	145	+0.25 points in somatic cell score and -0.96 pints in conformation final score.
16	6.08	0.07	6.77	6.71	291	+12.92 kg of milk fat and +9.81 kg of milk protein.
10	3.85	96.70	104.25	7.56	389	+562.53 kg of milk,+18.26 kg of milk fat and +14.63 kg of milk protein.
5	0.98	66.83	83.64	16.81	678	+1.59 points in conformation final score.

BTA: Bos taurus autosome, Mb: mega base.

In BTA2 ROH of 4.46 Mb showed the greatest effect on milk yield (+711.42 kg) and +18.32 kg of milk fat and +18.37 kg of milk protein. Only one ROH >16 Mb showed positive effect (+1.59) in conformation final score.

### ROH association with productive traits (milk, fat, protein), somatic cell score and conformation final score

From the total ROH, 17 were associated with productive traits. Table 4 shows ROH associated (p <0.05) with the PTA of milk, fat and protein yield and percentage, as well as conformation final score and that they were also present in more than 50 animals, in addition to the QTL and associations reported in the region.

**Table 4:** Quantitative trait loci and SNP markers identified in the ROH position associated to productive traits and conformation final score in this study.

BTA	Start Position (Pb)	Length (Mb)	Associated characteristics in this study	Associations and QTL
2	55,364	1.55	Milk fat percentage and Milk protein percentage.	20 Associations and 2 QTL (Conception rate, Length of productive life, Metritis, Milk caproic acid content, Milk caprylic acid content, Milk fat yield, Milk yield, PTA type, Rump width, Somatic cell score, Stature, Stillbirth, Udder attachment, Udder cleft, Udder depth, Udder height)
3	38,501	3.42	Somatic cell score, Milk fat yield, Milk fat percentage, and Conformation final score.	26 Associations and 2 QTL (Bone quality, Bovine tuberculosis susceptibility, Calving ease, Daughter pregnancy rate, Feet and leg conformation Foot angle, Inseminations per conception, Length of productive life, Milk fat percentage, Milk fat yield, Milk protein percentage, Milk protein yield, Milk yield, Net merit, Rear leg placement—rear view, Rear leg placement—side view, Somatic cell score, Stillbirth, Strength)
4	17,112	2.05	Somatic cell score, Milk fat yield, Milk fat percentage and Conformation final score.	8 Associations and 2 QTL (Angularity, Body condition score, Conformation score, Fat cover, Milk fat yield, Milk protein yield, Milk yield, Rump angle, Rump width, Stature)
6	36,766	5.21	Milk fat yield, Milk Production, Milk protein percentage and Conformation final score.	131 Associations and 19 QTL (Abomasum displacement, Body condition score, Body depth, Bovine tuberculosis susceptibility, Calving ease, Conception rate, Dairy form, Daughter pregnancy rate, Feet and leg conformation, Foot angle, Gestation length, Length of productive life, Milk alpha-S1-S2-casein percentage, Milk fat percentage, Milk fat yield, Milk iron content, Milk protein percentage, Milk protein yield, Milk riboflavin content, Milk tridecylic acid content, Milking speed, Net merit, PTA type, Rear leg placement—rear view, Retained placenta, Rump width, Semen volume, Somatic cell score, sperm counts, Sperm motility, Stature, Stillbirth, Stillbirth (maternal), Strength, Teat length, Teat placement–front, Teat placement–rear, Udder attachment, Udder cleft, Udder depth, Udder height)
9	28,755	1.38	Milk protein yield and Conformation final score.	1 Associations and 2 QTL (Clinical mastitis, Interval from first to last insemination, M. paratuberculosis susceptibility)
10	23,914	1.03	Milk fat yield, Milk protein percentage, and Conformation final score.	1 QTL (Milk yield)
11	39,943	5.67	Milk protein yield and Conformation final score.	107 Associations and 3 QTL (305-day milk yield Body depth, Bovine tuberculosis susceptibility, Calving ease, Carcass weight, Conception rate, Dairy form, Feet and leg conformation, First service conception, Foot angle, Inseminations per conception, Interval to first estrus after calving, Lean meat yield, Length of productive life, M. paratuberculosis susceptibility, Milk arachidonic acid content, Milk caproic acid content, Milk fat percentage, Milk fat yield, Milk glycosylated kappa-casein percentage, Milk iron content, Milk kappa-casein percentage, Milk protein percentage, Milk protein yield, Milk unglycosylated kappa-casein percentage, Net merit, Non-return rate, PTA type, Rear leg placement—rear view, Rump width, Somatic cell score, Sperm motility, Stature, Stillbirth, Strength, Teat length, Twinning, Udder attachment, Udder cleft, Udder depth, Udder height)
12	29,043	1.50	Somatic cell score, Milk fat percentage, Milk protein yield, Milk protein percentage and Conformation final score.	14 Associations and 4 QTL (Conception rate, Curd firming rate, Daughter pregnancy rate, First service conception, Length of productive life, Milk caproic acid content, Milk kappa-casein percentage, Milk protein yield, Milk unglycosylated kappa-casein percentage, Net merit, Somatic cell score)
15	74,750	1.37	Milk fat yield.	1 Associations and 1 QTL (Interval to first estrus after calving, Milk riboflavin content)
17	5,375	2.95	Milk yield, Milk protein percentage and Conformation final score.	38 Asocciations and 2 QTL (Calving ease, Conception rate, Dairy form, Daughter pregnancy rate, Dystocia, Feet and leg conformation, Foot angle, Inseminations per conception, Length of productive life, Milk alpha-casein percentage, Milk caproic acid content, Milk caprylic acid content, Milk fat percentage, Milk fat yield, Milk protein percentage, Milk protein yield, Milk yield, Net merit, PTA type, Rear leg placement—rear view, Rump angle, Stillbirth, Teat length, Teat placement–front, Teat placement–rear, Udder attachment, Udder depth, Udder height)
19	177,697	1.11	Somatic cell score and Conformation final score.	2 QTL (Somatic cell score, Milk protein percentage)
21	2,151,256	1.71	Milk fat yield, Milk fat percentage, Milk protein yield and Milk protein percentage	10 Associations and 2 QTL (Body weight, Milk kappa-casein percentage, Milk yield, Milking speed, Stillbirth)
23	15,894	1.54	Milk protein percentage and Conformation final score.	28 Associations and 2 QTL (Abomasum displacement, Calving ease, Dairy form, Daughter pregnancy rate, Dystocia, Foot angle, Inseminations per conception, Length of productive life, Milk fat percentage, Milk fat yield, Milk iron content, Milk protein percentage, Milk protein yield, Net merit, Rear leg placement—side view, Rump angle, Somatic cell score, Stillbirth, Stillbirth, Strength, Teat length)
25	81,699	2.19	Somatic cell score, Milk fat percentage, Milk yield, Milk protein yield, Milk protein percentage and Conformation final score.	33 Associations and 1 QTL (Calving to conception interval, Conception rate, Daughter pregnancy rate, Early embryonic survival, Fertilization rate, First service conception, Inseminations per conception, Interval from first to last insemination, Lean meat yield, Length of productive life, Methane production, Milk caproic acid content, Milk caprylic acid content, Milk fat yield, Milk protein percentage, Milk tetracosanoic acid content, Milk tricosanoic acid content, Milk tridecylic acid content, Net merit, Sire conception rate, Subcutaneous fat, Udder cleft)
26	756,489	2.19	Somatic cell score and Conformation final score.	61 Associations and 23 QTL (Anogenital distance, Aspartate aminotransferase level, Body depth, Calving ease, Conception rate, Dairy form, Daughter pregnancy rate, Feet and leg conformation, Foot angle, Length of productive life Milk C14, C16, C18 index, Milk capric acid content, Milk caprylic acid content, Milk decenoic acid content, Milk fat percentage, Milk fat yield, Milk lauric acid content, Milk lauroleic acid content, Milk myristic acid content, Milk myristoleic acid content, Milk palmitic acid content, Milk palmitoleic acid content, Milk protein percentage, Milk protein yield, Milk stearic acid content, Milking speed, Net merit, PTA type, Rear leg placement—rear view, Rear leg placement—side view, Rump angle, Rump width, Somatic cell score, Stature, Stillbirth, Strength, Teat length, Teat placement–rear, Udder attachment, Udder cleft, Udder depth)
27	18,888	5.36	Milk yield, Milk protein percentage and Conformation final score.	287 and 28 QTL (Bovine tuberculosis susceptibility, Calving ease, Cell-mediated immune response, Conception rate, Dystocia, Foot angle, Gastrointestinal nematode burden, Milk fat yield, Milk protein percentage, Milk protein yield, Milk tetracosanoic acid content, Milk tridecylic acid content, Milk yield, Milk zinc content, Net merit, Percentage abnormal sperm, Rear leg placement—side view, Rump angle, Somatic cell score sperm counts, Sperm motility, Stature, Strength)
29	108,487	8.28	Somatic cell score, Milk yield, Milk protein yield and Conformation final score.	26 Associations and 1 QTL (Conception rate, Daughter pregnancy rate, Hip height, Inseminations per conception, Interval to first estrus after calving, Lean meat yield, Milk fat yield, Milk glycosylated kappa-casein percentage, Milk kappa-casein percentage, Milk tridecylic acid content, Milk unglycosylated kappa-casein percentage, Number of embryos, Rump angle, Somatic cell score)

BTA: Bos Taurus Autosome, Pb: Pares de bases, Mb: Mega bases.

Runs Of Homozygosity associated with conformation and producction traits (P < .05), in more of 50 animals.

The ROH associated with the productive and conformation traits were present in 1,847 animals, and most of them had a length shorter than 5 Mb. Eight were in the range of 1 to 2 Mb in length, four in the range of 2 to 3 Mb, one in the range of 3 to 4 Mb, three in the range of 4 to 5 Mb and one of 8.28 Mb.

Some ROH were associated with several traits and others only with one, for example in BTA 15 a ROH of 1.37 Mb of length present in 108 animals were associated with milk fat yield only.

In BTA 6, a ROH of 5.21 Mb of length was found present in the largest number of animals (257), which was associated with milk fat yield, milk production, milk protein percentage and conformation final score.

The longest ROH, 8.28 Mb of length in BTA 29, was present in 65 animals and found associated with somatic cell score, milk yield, milk protein yield and conformation final score.

The ROH with the most associations was found on BTA 25 in 82 animals, had a 2.19 Mb length and was found associated with somatic cell score, milk fat percentage, milk yield, milk protein yield, milk protein percentage and conformation final score.

## Discussion

Purfield et al. [1] showed that the number of ROH per chromosome was higher in long BTA and it decreased according to the length of the chromosome; for example, in chromosome 1 they found a total of 11,513 ROH in 867 bovines of different breeds (Angus, Holstein, Friesian, Simmental, Limousine, Charolaise, Hereford and Belgian Blue) which represented 12.39% of the chromosome covered by ROH; similar to the finding in this study in which 27,711 ROH were found on the same chromosome, which represented 11.31% of the total length of the chromosome covered by ROH.

The average number of ROH per animal in this study was 71.38 ± 0.77, a result that showed similarities with the report by Szmatoła et al. [27] who found a ROH average per animal (78.8 ± 9.6) in Holstein cattle from Poland which included animals with 46,948 SNPs; differing with the findings in Holstein cattle from North America in which the average was 82.3 ± 9.83 ROH per animal [15]. Another study performed in US Holstein cows by Kim et al. [14], in which they included animals with 41,950 SNP, found an average of 41.8 ROH per animal and an average length of 6.61 Mb, longer than the calculated in this work (4.80 Mb).

Differences could be explained by the selection pressure applied on each population, as well as the number of SNPs included in each study.

Goszczynski et al. [28] published a study carried out in inbred and outbred bulls of the Retinta cattle from Spain in which they found, similar to this study, the length ROH with greatest presence were those of 1 to 2 Mb (>50%) in both groups of animals (inbred and outbred). The average length in the population of Retinta cattle was of 3.51 ± 0.08 Mb in inbred bulls, less than that reported in this study of the Mexican Holstein population (4.80 ± 0.77 Mb). Differences could be explained by the number of animals in each study or by selection pressure in each population, considering a lower pressure in the Retinta breed of Spain.

Forutan et al. [15], demonstrated that the more abundant ROH in US Holstein cattle were those with less than 2 Mb, representing 43.5%, followed by those of 2 to 4 Mb with 23.9%. Marras et al. [29] published a study carried out in Holstein cattle from Italy, which showed similarities with the study of Forutan et al. [15], where the ROH with less than 2 Mb represented 56.9%, followed by those of 4 to 8 Mb with 20.8%; these studies showed similarities to the results obtained in this study, since the ROH from 1 to 2 Mb were the most frequent (51.33%) and the ROH of 2 to 4 Mb had a frequency of 22.33%. The frequency of ROH >16 Mb in this study (3.53%) was also similar to those from other results in Holstein cattle [15, 29], who reported 4.7% and 3.7% in the same length category, results that could demonstrate that the Mexican Holstein population present an ancient inbreeding equal the other populations.

The average length of the genome covered by ROH was 276.89 Mb for this population; a length similar than that reported in Holstein cattle from Italy, which was 297 Mb [29] with a Froh of 3.8 ± 2.1%, slightly above the one reported in this study. In the US Holstein cattle, two studies also demonstrated similar lengths of the genome covered by ROH to this study, which were 290.6 and 299.6 Mb [15, 24]. Comparing those results with the ones obtained in this study, one can assume that the selection intensity in the Holstein cattle is different in the populations, although reproductive and genomic technologies have dispersed globally genetic material.

The chromosome 20, presented the highest proportion covered by ROH (18.78%), due to its average length of ROH (6.34 Mb), followed by chromosome 10 with 16.76% with average length of ROH of 5.88 Mb. This possibly is due to the fact that genes within these BTA encode for economically important traits and may have been heavily selected for. Szmatola et al. [27] showed results of ROH in Holstein cows from Poland, where the BTA with the highest proportion covered were the 10, 16 and 20, showing a greater trend of selection related with these regions.

Although most of the ROH associated to production traits were found at the beginning of the BTA (within the first 1.5 Mb), with lengths ranging from 1.03 Mb on BTA 10 to 8.28 Mb on BTA 29, ROH distribution was found scattered throughout the BTA. This could be affected by many factors, such as that BTA in cattle are acrocentric [30], the length of the ROH and the selection objectives in the animals [31], and the low recombination at the ends of BTA [32], although some studies have reported a higher frequency of ROH in the middle of BTA, where low recombination rates have been reported [33].

The ROH with positive or negative effects on the production traits adjusted to 305 days, were different from the ROH associated with the different PTA, this is due to the fact that in the analyzes of the PTA, the effects of each one SNP present in the ROH are taken into account and in the analyzes of traits adjusted to 305 days, only the presence or absence of the ROH in the animals is taken into account.

In a study carried out in Canadian Holstein, Makanjuola et al. [24] identified ROH on BTA 8, 13, 14 and 19, unfavorably associated with production and fertility traits. They estimated that ROH with decreased milk production by 247.30 kg, fat production by 11.46 kg and protein production by 8.11 kg. In this study, in BTA13, a ROH (1.52 Mb) was found in a different position than in the Makanjuola study, which showed positive effects on milk production +299.02 Kg.

The length of 22 ROH with effects on the productive traits was found from 1 to ~4 Mb and only three ROH with length greater than 6 Mb was found. Makanjuola et al. [24] reported that the length of the ROH with negative effects on the productive traits was from 1 to ~4 Mb.

In BTA 2, a ROH of 1.55 Mb was detected in this study associated a Milk fat percentage and milk protein percentage, in a region that includes annotations of 2 QTL and 20 associations with milk yield, length of productive life and several other traits like udder cleft, udder depth, and udder attachment in the same breed [34].

One ROH in BTA 6 with a length of 5.21 Mb and associated with fat, protein and milk yield in this study, included annotations presented by Schrooten et al. [35] who reported associations of different QTL with milk fat amount in Holstein cattle from Netherlands. In the same region Cole et al. [34], reported multiple associations and QTL related to conformation and productive traits in US Holstein cattle.

For one ROH in BTA 15 with a length of 1.37 Mb, Poulsen et al. [36] reported in the same location in Danish Holstein cows, the presence of a QTL associated to milk riboflavin content. Furthermore Daetwyler et al. [37] reported in the same location a QTL for Interval to first estrus after calving in Canadian Holstein.

The ROH in BTA 14 associated to conformation final score in this study presented other associations with milk yield, fat, protein and somatic cell score, and furthermore it has annotations related to conformation traits, production and other trait related to animal functionality. In a ROH located on BTA 19 (1.11 Mb), a QTL related to milk somatic cell score and milk protein percentage was reported in German and French Holstein cattle [38]. On BTA 6, in a ROH of 5.21 Mb, 19 QTL were found and 131 associations with body conformation, length of productive life, milk components and fertility traits were reported [29].

According to Kim et al. [14] who demonstrated the existence of signatures of selection on BTA 2, 7, 10, 20 and 24 associated with milk, fat and protein yield, the ROH could be used as a selection tool because the loss of important alleles for milk yield could be minimized when selection is focused on few functional traits. It is important to consider the possible drawbacks of using ROH as selection criteria, because undesirable traits could be selected for; for example, in the ROH region on chromosome 27 (5.36 Mb) associated with conformation final score, milk yield and protein percentage, even though many desirable traits have been reported, undesirable traits like a QTL associated to Bovine tuberculosis susceptibility [39] and dystocia are also found [40].

A possible solution to this problem could be the use of duplicate ROH with different lengths, avoiding the inclusion of undesirable characteristics or the identification of the animals that carry these ROH and mate with non-carrier animals in order to reduce their frequency in the population.

## Conclusion

In this study, ROH with lengths between 1 to 4 Mb were found associated with productive traits with positive and negative effects on production. A large number of QTL and associations related to the productive traits evaluated were also reported within the ROH locations, so it is concluded that these could be used in the evaluation and selection of Holstein cattle for genetic improvement.
